# Myocardial phenotypes and dysfunction in HFpEF and HFrEF assessed by echocardiography and cardiac magnetic resonance

**DOI:** 10.1007/s10741-019-09880-4

**Published:** 2019-11-14

**Authors:** Bostjan Berlot, Chiara Bucciarelli-Ducci, Alberto Palazzuoli, Paolo Marino

**Affiliations:** 1 Department of Cardiology, University Medical Centre Ljubljana, Zaloska cesta 7, 1000 Ljubljana, Slovenia; 2 Bristol Heart Institute, Bristol National Institute of Health Research (NIHR) Biomedical Research Centre, University Hospitals Bristol NHS Trust and University of Bristol, Bristol, UK; 3 Cardiovascular Diseases Unit Department of Internal Medicine, Le Scotte Hospital University of Siena Italy, Siena, Italy; 4 Department of Translational Medicine, Eastern Piedmont University, Via Solaroli 17, Novara, Italy

**Keywords:** Echocardiography, Cardiac magnetic resonance imaging, Ischemic and non-ischemic heart failure, Systolic and diastolic dysfunction

## Abstract

Heart failure (HF) with either reduced or preserved ejection fraction is an increasingly prevalent condition. Cardiac imaging plays a central role in trying to identify the underlying cause of the underlying systolic and diastolic dysfunction, as the imaging findings have implications for patient’s management and individualised treatment. The imaging modalities used more frequently in patients with heart failure in clinical routine are echocardiography and cardiac magnetic resonance. Both techniques keep some strengths and weakness due to their spatial and temporal resolution. Notably, several features in the diagnostic algorithm of heart failure with preserved systolic function (HFpEF) may be improved by an integrated approach. This review focuses on the role of each modality in characterising cardiac anatomy, systolic and diastolic function as well as myocardial tissue characterisation in the most common phenotypes of dilated and hypertrophied hearts.

Cardiac imaging plays a central role in trying to phenotype the underlying cause of heart failure (HF) which has implications for patient’s management and individualised treatment. Heart failure has recently been classified as heart failure with reduced ejection fraction (HFrEF) or preserved ejection fraction (HFpEF) [1]; although, it is still debated whether these are two different entities or just a different stage of same ongoing process [2, 3]. The clinical imaging modalities available to investigate patients with heart failure are echocardiography, nuclear cardiology techniques, computed tomography and cardiac magnetic resonance (CMR). In this paper, we review the role of echocardiography and CMR in patients with HFrEF and HFpEF.

Two-dimensional transthoracic echocardiography is the first line imaging tool providing information on function, cavity size, relative wall thickness (RWT) and myocardial mass which are used for classification of typical geometric phenotypes—concentric or eccentric hypertrophy and remodelling [4, 5]. Moreover, echocardiography deformation imaging can discriminate between active myocardial segmental deformation and passive displacement of a dysfunctional myocardial segment due to adjacent segment tethering and global cardiac motion. However, echocardiography alone cannot identify interstitial infiltration and intracellular accumulation of metabolic substrates [6].

Cardiac magnetic resonance (CMR) is the gold standard for cardiac anatomical and functional quantification, with unique capabilities of non-invasive tissue characterisation [7–9], complementing well echocardiography. Cine imaging covering the LV in short axis from apex to base is used for measuring left ventricular (LV) volumes, ejection fraction (EF) and regional function. The 3D dataset is not affected by geometric assumptions and therefore less prone to error compared with two-dimensional (2D) echocardiography particularly in remodelled ventricles [10]. Non-invasive tissue characterisation by CMR can be achieved with T2 imaging and T1 imaging post-contrast injection. The latter technique, called late gadolinium enhancement (LGE), relies on gadolinium-based contrast agents temporarily accumulating in regions of increased extracellular space (due to myocardial scar, necrosis, fibrosis, infiltration), following the underlying pathophysiological process, and creating typical LGE patterns [11] that guide the diagnosis.

Novel CMR tissue characterisation techniques are called CMR relaxometry (T1 and T2 mapping and extracellular volume fraction (ECV)) which allow a more detailed and quantitative approach to tissue characterisation and 4D-Flow which provides quantitative information on intracavitary flows. Current applications appear particularly useful for diastolic dysfunction detection although they deserve a specific comparison with traditional Doppler and Tissue Doppler analysis in order to confirm the applicability in clinical practice.

The most common cardiomyopathic processes underpinning HFpEF (hypertrophied phenotypes) and HFrEF (dilated phenotypes) are discussed below.

## Left ventricular hypertrophic phenotypes

Left ventricular (LV) hypertrophy (LVH) is a consequence of an underlying genetic or acquired condition and it is accompanied by alterations in cardiac function and haemodynamics. The differential diagnosis between LVH due to physiological adaptation or underlying pathology can be challenging.

Non-physiological left ventricular hypertrophy (LVH) regardless to the underlying cause leads to important cardiovascular complications such as atrial fibrillation, diastolic and systolic heart failure [12], and it is associated with increased risk for all-cause morbidity and mortality [13].

### Hypertensive heart disease

Systemic blood pressure elevation is the most common cause of the increment in ventricular mass with a high RWT, which results in concentric or eccentric hypertrophy and concentric remodelling [14]. These structural changes provide a mechanism for maintenance of normal LV systolic wall stress in the presence of a high systolic pressure, although up to 60% of the variance of LV mass may be due to genetic factors independent of blood pressure [15]. The earliest change appears to be an increase in RWT before there is a detectable increase in LV mass [16]. In hypertensive heart disease (HHD), the wall thickening is influenced by ethnicity, neurohumoral factors and genetic variants, is commonly symmetrical and in basal segments rarely exceeding 15 mm [17]. However, locally increased wall stress in the basal septum can result in regionally increased wall thickness—also known as septal bulging, mimicking other asymmetric hypertrophic phenotypes. Clinical heart failure in hypertensive heart disease can occur either in the setting of reduced or preserved LVEF [18]. Echocardiography is the first line diagnostic tool. However, traditional parameters offer little clue about the underlying aetiology, hence novel applications are gaining in popularity. Despite that cardiac hypertrophy may be analysed with the two methods, there is an existing gap in LV hypertrophy and mass measurement between the two techniques: indeed LV mass is currently calculated with standardised formulas in a parasternal longitudinal view axis in echo by the assumption that LV has an ellipsoid shape [19]. Conversely, LV mass in CMR is measured in a transversal view by the sum of the whole myocardial slices from basal to apical level [20]. This make CMR much more reproducible without geometric assumption and lower cut-off value compared with echo. This is an important item when physicians calculate the global CV risk in hypertensive or high-risk subjects.

Global longitudinal strain (GLS) is an emerging parameter currently available with both methods. GLS is typically reduced in advanced stages of HHD, and it is strongly associated with diastolic dysfunction, it is less dependent on afterload changes and degree of LVH compared with EF, and it has a role in differentiating HHD from other hypertrophic phenotypes [21, 22]. CMR is superior to echocardiography due to its possibility of tissue characterisation. By magnetic resonance study, it is possible to ascertain the burden of myocardial fibrosis, extracellular collagen deposition extension and site. Therefore, the absence of LGE on CMR increases the predictive power in diagnosing HHD over and above geometrical and morphological features [23, 24]. Finally, HHD is normally accompanied with structural or functional changes in arteries or end organs (heart, blood vessels, brain, eyes and kidney) as a consequence of long lasting hypertension even in asymptomatic patients [25]. Evidence of increased blood pressure is the clinical hallmark of the disease, even though approximately 15% of patients might present with a normal office BP (masked hypertension) [26]. The most common functional feature of HHD, apart structural LVH and remodelling, is the alteration of left filling pressure due to both increased LV mass and stiffness associated with reduced elastic properties of myocardial tissue. Notably, echocardiography and Doppler application due to its high temporal resolution, offers a suitable and practical tool to recognise LV diastolic filling dysfunction by the combined analysis of trans-mitral pulsed waves Doppler and myocardial tissue Doppler (TDI). Therefore, additional study of pulmonary vein flow allows to a characterisation of diastolic degree and haemodynamic conditions into the left cardiac chamber [27, 28]. A similar analysis have recently been reproduced during CMR examination in a phase-contrast acquisition measuring trans-mitral and venous flow velocities in a post-processing analysis including around 40 cardiac phases of the whole cardiac cycle. Additional analysis comprises myocardial tagging that calculates radial and circumferential motion during cardiac relaxation, and more recently, feature tracking to detect myocardial strain [29].

### LV hypertrophy in cardiac amyloidosis

Infiltrative myocardial disease particularly cardiac amyloidosis can present with very heterogenous hypertrophic phenotypes with wide ranges of wall thicknesses including normal [30]. Granular appearance on echocardiography, biventricular hypertrophy with involvement of the right ventricular free wall, increased thickness of the atrio-ventricular valves, thickening of the interatrial septum and the presence of a small pericardial effusion usually trigger a suspicion [30]. Whereas the light chain amyloidosis (AL) normally follows concentric symmetric hypertrophic pattern, the transthyretin amyloidosis (TTR) is typically asymmetrical [31]. Thickened myocardium and conventional echo parameters have low accuracy for the diagnosis of cardiac amyloidosis, which is mostly due to their low sensitivity [32]. However, some echo indices, enlarged left atrial volume index, reduced LV diameters and volumes, global parietal thickness and increased parietal backscatter have higher specificity and have a potential to be used to ‘rule in’ potential amyloidosis cases [33, 34], suggesting diastolic dysfunction as a leading functional abnormality in this population. A typical restrictive filling Doppler pattern is characterised by a high early wave with shortened deceleration time and isovolumetric time. Conversely, the atrial contribution is reduced and often blunted even in those patients without atrial fibrillation. Pulmonary flow analysis demonstrates increased atrial reverse wave associated with both reduced systolic and diastolic waves [35]. The alteration of intrinsic myocardial relaxation forces is demonstrated by significative lowering of tissue *e*′ velocity associated with increased *E*/*e*′ ratio. Myocardial deformation parameters or patterns of regional strain values such as relative apical sparing or septal apical-base longitudinal strain gradient may have a better differentiating capacity in detecting and differentiating cardiac amyloidosis from other hypertrophic substrates, including HCM, hypertrophy in aortic stenosis or metabolic cardiomyopathies [36]. Using deformation imaging, LV dysfunction can be detected prior to any signs of morphological or functional impairment as assessed by 2D echocardiography or assessment of diastolic function [34]. However, all aforementioned conditions may present with regional impairment patterns that closely resemble cardiac amyloidosis. CMR is superior to echocardiography and plays an important role in diagnosis of cardiac amyloidosis due to peculiar myocardial and blood-pool gadolinium kinetics in this disease. Detection of transmural or subendocardial LGE with suboptimal myocardial nulling caused by similar myocardial and blood T1 signals, is unusual but highly specific pattern in amyloidosis. Furthermore, native T1 mapping values and ECV are severely increased in amyloidosis [37]. These novel CMR parameters are more sensitive for detecting early disease than LGE imaging, correlate better with markers of systolic and diastolic dysfunction and have a potential to give information on cardiac amyloid load without usage of contrast agents [38].

## Left ventricular dilated phenotypes

Dilated phenotypes are a heterogenous group characterised by large LV cavities with eccentric remodelling or hypertrophy and impaired contractility. Such phenotypes can be a response to abnormal loading conditions typically in valvular disease or hypertension, severe coronary or congenital disease [39] or predominantly confined to heart muscle like in inherited or acquired cardiomyopathies. Transthoracic echocardiography is used as a first line imaging tool for identifying and description of the phenotype. It typically shows global left or biventricular hypokinesis with or without regional wall motion abnormalities. Ventricular and atrial dilatation, intracardiac thrombi and functional mitral regurgitation due to annular dilatation might also be noted. Doppler parameters can assist in quantifying valvular abnormalities and the severity of diastolic dysfunction [40]. CMR is superior to echocardiography, it provides accurate assessment of ventricular volumes, wall thickness and contractile function, as well as tissue characterisation [41]. Typical patterns of subendocardial, mesocardial and subepicardial LGE distribution reflect underlying pathophysiological processes and reveal aetiology with such a level of confidence that in many cases myocardial biopsy may be omitted. CMR relaxometry can indicate myocardial necrosis, scarring, focal and diffuse replacement interstitial fibrosis, whereas high signal T2 intensity or increased T2 mapping values suggest myocardial oedema and inflammation [42]. The 4D-Flow technology allows the assessment of flow-based forces and their altered haemodynamic effects on the myocardial wall with a potential to become a maker of progressive adverse cardiac remodelling [43].

### Post-ischemic heart failure

Reflecting perfusion contraction matching and mismatching, ischaemic heart failure (IHF) consists of a spectrum of pathophysiological states, from early remodelling characterised by wall thinning and dilatation to irreversible late remodelling resulting from myocardial fibrosis and scar [44]. Echocardiography with three-dimensional (3D) acquisition, in addition to stress and contrast echocardiography enables a comprehensive view of myocardial function, contractile reserve and perfusion. Resting two-dimensional echocardiograms are the first step in IHF patients and allow us to quantify LV function and assess the presence of resting regional wall motion abnormalities, which are the hallmark of disease [45]. Use of myocardial deformation imaging enables detection of abnormal myocardial contractility in earlier stages of the disease, but it is of limited value in defining aetiology or predicting viability of myocardium [46]. CMR on the other hand has a saying in myocardial viability by identifying partial or full thickness myocardial infarction based on the pattern of LGE distribution - subendocardial or transmural, respectively. Moreover, CMR showed to be superior to echocardiography in discriminating between hibernating and necrotic myocardium independently from wall thickness [47]. Akinetic segments with > 50% transmural LGE are considered non-viable (necrotic), whereas akinetic segments with no LGE have an approximately 80% likelihood of functional recovery [48]. Once the scar transmurality exceeds 50% the likelihood of functional recovery drops to approximately 8% [49]. The presence and amount of LGE is prognostically important and associated with major adverse cardiac event (MACE) and cardiac mortality [50], and it is an independent predictor of all-cause mortality or cardiac transplantation [51].

### Idiopathic dilated cardiomyopathy

**Fig. 1:**
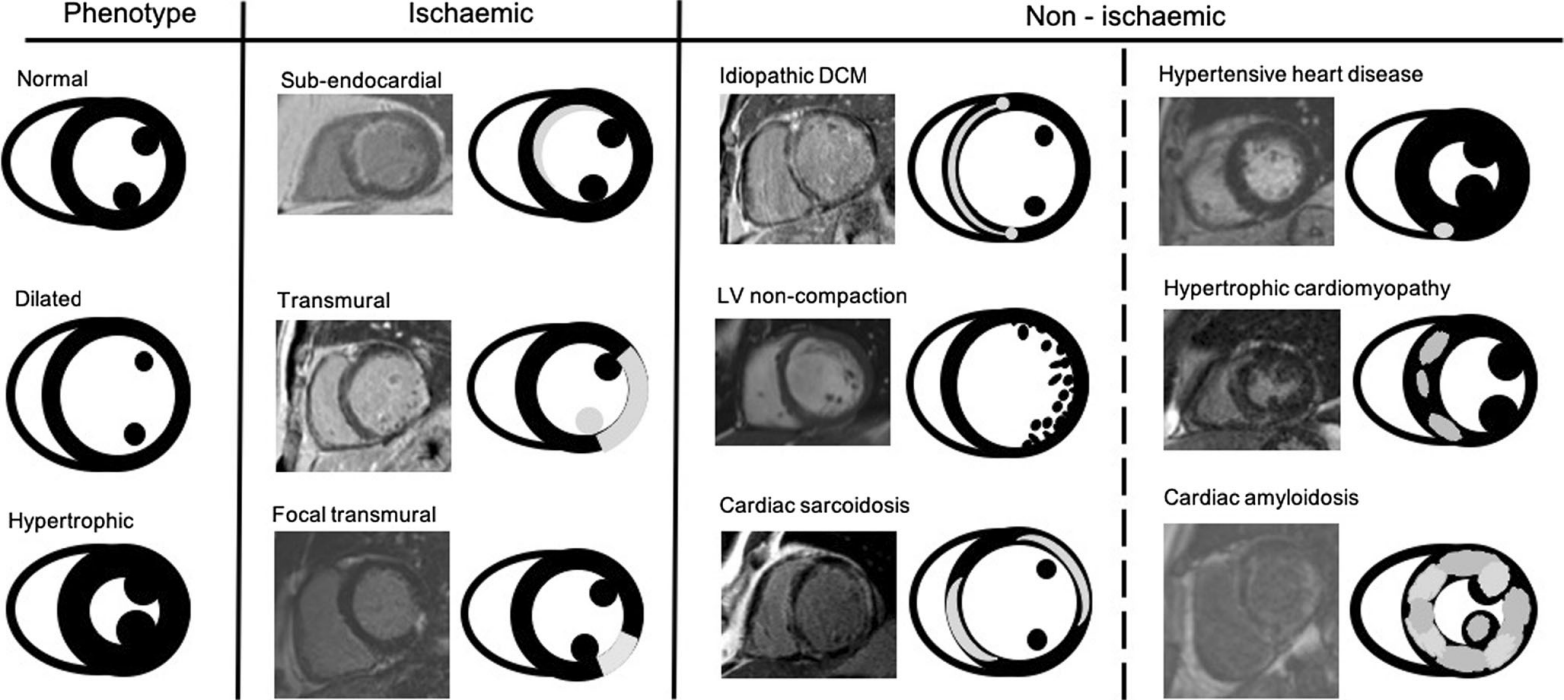
Patterns of late gadolinium enhancement and corresponding cardiac MRI images

Mutations in over 50 genes have been associated with dilated cardiomyopathy [52]. Multiple failing mechanisms result in altered force generation and cell death, leading to left ventricular systolic dysfunction and heart failure. Diagnostic criteria of LV dilatation with reduced function applies to ‘idiopathic’ DCM once secondary causes have been excluded [53, 54]. Echocardiography is the first-line diagnostic tool. Volumes and EF acquired from 3D echocardiography correlate better with CMR and their use is recommended when feasible [55]. LV enlargement and alterations in GLS typically precedes LV dysfunction assessed by 2D classical parameters [56]. Moreover, decreased GLS showed ability to discriminate gene-positive phenotype-negative individuals from normal controls, which may permit early institution of therapy for genetic DCM [57]. Similar findings should be expected from the feature tracking obtained by CMR. CMR plays a central role in phenotypic assessment. Approximately 25% of patients with dilated cardiomyopathy (DCM) will have evidence of mid-wall fibrosis [58] which is an independent predictor of mortality and morbidity. DCM patients with mid-wall fibrosis had a similar outcome to those with ischemic disease [59]. Thus, as with ischemic cardiomyopathy, the presence of fibrosis/scar is a marker of adverse outcome and worse response to device therapy. Similarly, T1 mapping sequences together with measurement of ECV expansion has proved to have an additional value over EF in prognostic assessment (Fig. 1) [60].

## Systolic dysfunction in HFpEF and HFrEF patients

The longitudinal trajectories of LV ejection fraction vary between the two conditions in a very sensitive way. When adequate follow-up is available, repeat echocardiograms in HFpEF can detect a maximum of 2–5% fell in EF over 5 years, with a larger fall in the presence of coexisting coronary artery disease [61, 62]. A similar analysis, conducted over a follow-up period up to 15 years in HFrEF patients, observed an inverted U-shaped relation whereby EF indeed increased over the initial 10 years but slowly declined afterward [63]. Comparable data should be available when looking at systolic deformation, particularly in the circumferential or radial direction, either assessed using CMR or echocardiography [64]. Thus, clear discrepancies in LV systolic performance between HFpEF vs. HFrEF populations long-term trajectories make unlikely a mechanistic continuum between the two conditions, which can be represented as distinct HF phenotypes [65].

## Diastolic dysfunction in HFpEF and HFrEF patients

More similarities, than discrepancies, instead, can be detected when looking at diastolic dysfunction in HFpEF vs. HFrEF patients. Diastolic dysfunction contributes to exercise intolerance, both in systolic and primary diastolic dysfunction. In both conditions, in fact, diastolic impairment limits exercise tolerance before resulting in symptoms at rest [66].

In the absence of mitral stenosis, the two major factors that determine the early diastolic mitral valve pressure gradient and the rate of LV filling are the rate of LV relaxation and the LA pressure at the time of mitral valve opening [67]. With exercise, normally, there is a fall in early diastolic LV pressure that results in an increased early diastolic pressure gradient, without an increase in LA pressure to abnormal levels. This increment in gradient, obtained with no increment in LA cavity pressure, produces an improvement in the rate of early diastolic LV filling [68]. When HF ensues, however, peak early diastolic filling rate and diastolic mitral valve pressure gradient increase as during normal exercise but with a different mechanism. Relaxation, in fact, which is slower at rest in HF, is further slowed down during exercise, while early diastolic LV pressure increases [69]. Thus, the increased rate of early diastolic LV filling and the mitral valve pressure gradient in HF result from an abnormal increase in LA pressure. Non-invasive methods to identify LV and LA pressure have recently been discussed and even the traditional echo parameters considered markers of elevated LV filling pressure such as reduced mitral deceleration time (DT) and isovolumic relaxation time (IVRT) blunted A wave and high *E*/*e*′ ratio have been debated. In theory, the occurrence of the above-mentioned picture is typical of a restrictive pattern and along with increased LA volume and increased pulmonary pressure, reflects high filling pressure in either HFrEF and HFpEF [70]. However, only a few studies compared Doppler and CMR parameters with direct haemodynamic measurement. Most of these studies analysed only *E*/*e*′ ratio in relation to wedge pressure, but a complete measurement of left chambers pressure is lacking [71, 72]. Therefore, correlation between *E*/*e*′ ratio and LVEDP in a recent metanalysis appears modest and patients with atrial fibrillation (AF) were excluded [73]. All these concerns put some doubts and limitation in evaluation of isolated diastolic dysfunction and consequently in the diagnosis of HFpEF. Thus, TDI and PW Doppler parameters may be integrated with careful analysis of cardiac structure and LV remodelling. Accordingly, elevated LA pressure leads to LA dysfunction and remodelling being commonly observed in both phenotypes. These findings, at a difference from systolic properties, would suggest a mechanistic continuum, as far as diastole is concerned, between the two conditions. It must be recognised, however, that LA remodelling in HFpEF and HFrEF, has been reported as being slightly different, with more dilation and systolic dysfunction in HFrEF and with increased stiffness, pulsatility and predilection for atrial fibrillation (AF) in HFpEF [74]. We think that this distinction is partly artefactual. The extent of LA pulsatility, that is a direct function of the stiffness of the cavity, may contribute to the pulsatile major component of right ventricular afterload [75]. Thus, from this perspective, diastolic LA dysfunction can be seen as an active contributor to symptoms and to disease progression for both phenotypes.

## Novel methods combining echo and CMR analysis for best risk prediction in atrial fibrillation patients

Diastolic dysfunction is a dominant feature in many HF patients. LV diastolic dysfunction causes LA dilatation, which can lead to AF [76]. Despite of being advance in management and treatment, AF remains a source of considerable morbidity and mortality worldwide. For the patients in sinus rhythm LV, filling pressures and diastolic function grade can be determined reliably by a few simple echocardiographic or CMR parameters with a high feasibility [77]. However, for the patients in AF regardless whether AF is a reason or consequence, assessment of diastolic function is challenging. Assessment in this condition in limited by cycle length variability, absence on an organised atrial activity and frequent occurrence of atrial enlargement regardless of filling pressures [78]. Recently, some new indexes using speckle tracking echocardiography have shown promising results showing the association between LV systolic and diastolic strain, LA strain and LV diastolic function in AF patients [79]. Indexes potentially capable of describing the delicate atrio-ventricular relation should convey the strongest pathophysiological information and be less influenced by R-R variations, if they can be comprehensively acquired in 1 single beat.

**Fig. 2:**
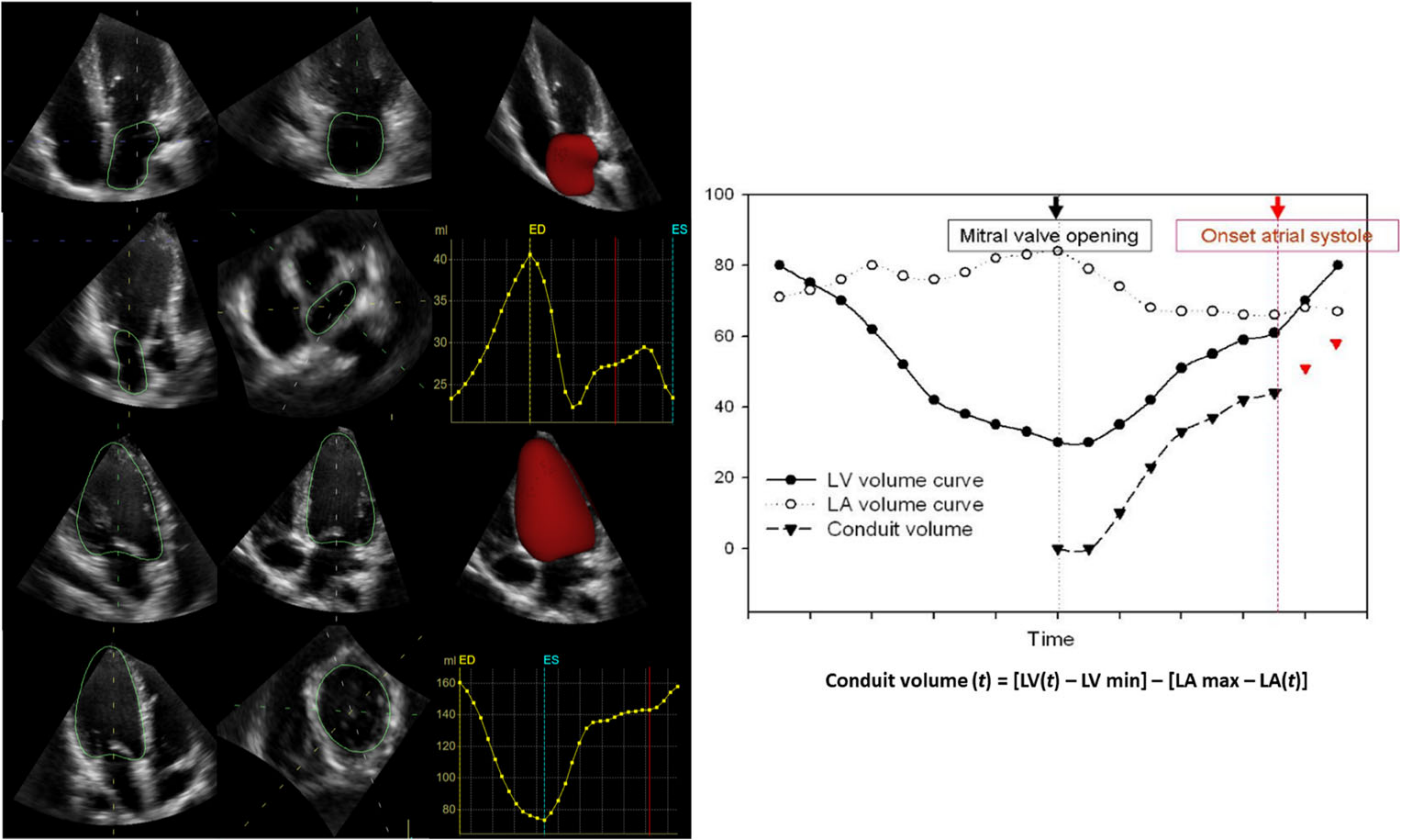
Left atrial conduit volume quantification by 3D echocardiography. *Left*, single-beat simultaneous left atrial (LA)–left ventricular (LV) pyramidal 3D echocardiographic full-volume dataset as obtained from the apex in a given patient, using the 4V transducer during held respiration (frame rate > 16.5/s).The volume data are displayed in real time, three apical views and one cross-sectional slice, with optional volume-rendering techniques for visualisation of valves and structures. *Right,* conduit volume is quantified according to the formula: (LV at time (*t*) minus LV minimum) minus (LA maximal minus LA at time (*t*)), integrating volume data from minimum LV volume to the beginning of LA contraction (as identified from simultaneously acquired ECG signal) and expressed as percent of LV stroke volume

Recently, in characterising the governing role of the four-chamber (near) constant-volume pump physiology, wherein the atrial and ventricular volumes simultaneously reciprocate throughout the cardiac cycle, CMR has elucidated and characterised LA and LV phasic function, thereby quantifying the conduit contribution to ventricular filling as the integral of net, diastolic, instantaneous difference between synchronised atrial and ventricular volume curves [80]. Because cardiac CMR availability is limited, 3D echocardiography can be employed to acquire complete and simultaneous LA and LV full-volume datasets to characterise the volume of both left-sided cardiac chambers at each time point during the cardiac cycle in order to quantify conduit [81] (Fig. 2).

Using single-beat simultaneous left atrial and ventricular full-volume 3D dataset, it was demonstrated that the atrial conduit contribution to ventricular filling has a direct relationship with the degree of underlying ventricular diastolic impairment in HF patients [82]. More recently, it was shown that conduit quantitation is also able to predict 1-month AF recurrence in a population of persistent AF patients imaged immediately after and/or before electrical cardioversion [83]. These findings support the concept that conduit (independently of the imaging technique used to quantify it), reflects intrinsic atrial pathology that cannot be sufficiently explained by ventricular pathology only and thus it could be proposed as a clinically effective tool for exploring the link between AF and diastolic dysfunction, in excess of ventricular derangement.

**Fig. 3:**
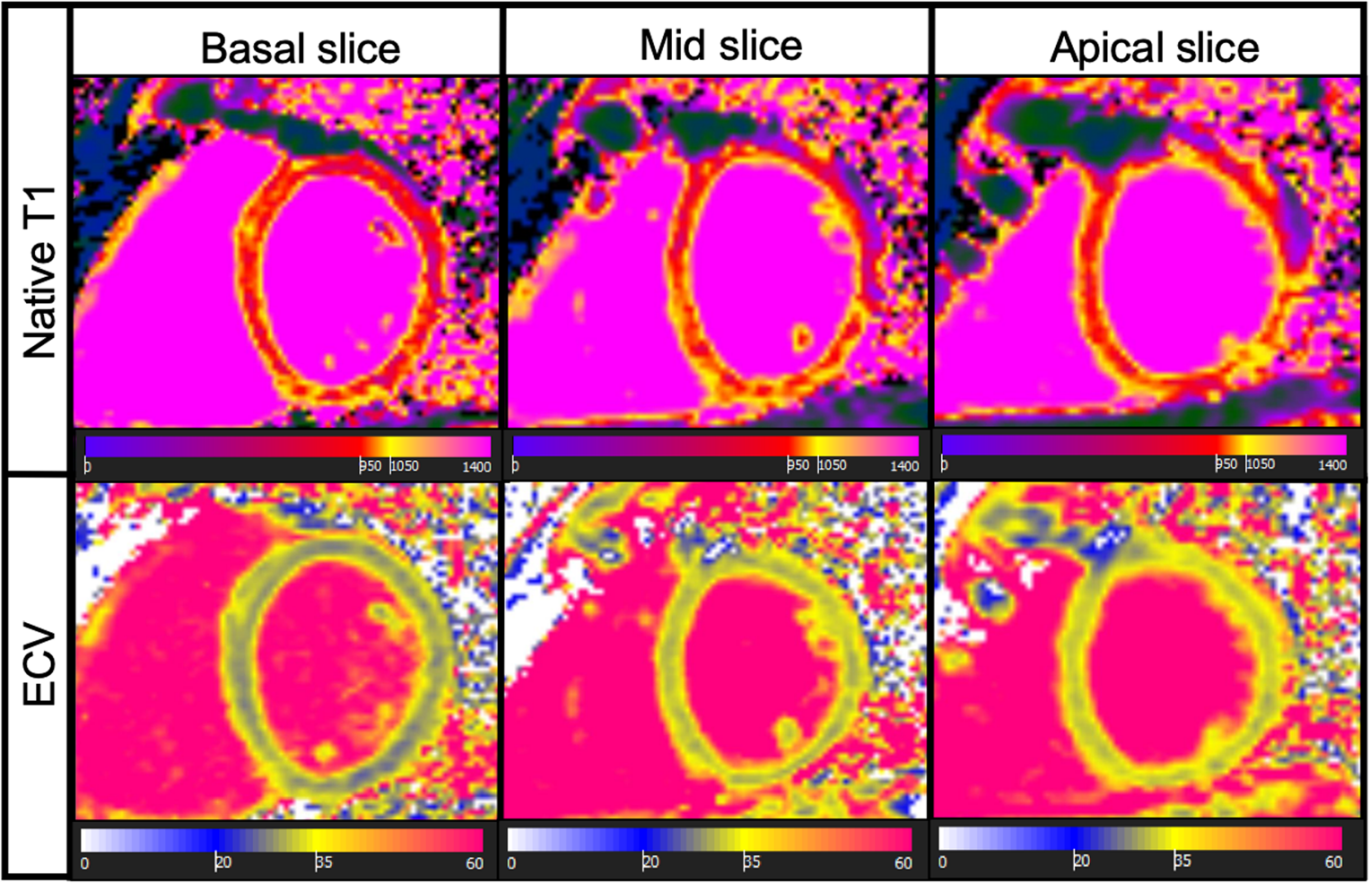
Assessment of myocardial fibrosis by CMR. Basal, mid- and apical slices showing native T1 (*top line*) and ECV (*bottom line*) colour maps used for quantitative assessment of cardiac fibrosis

In addition to LA and LV volume curves, CMR can provide further information on myocardial tissue characteristics. Myocardial fibrosis have been implicated in the pathophysiology of HFpEF by promoting adverse ventricular remodelling, increasing myocardial stiffness, and in turn, causing diastolic dysfunction [84]. Diffuse interstitial fibrosis, a precursor for replacement fibrosis, is not detected by LGE but correlates with T1 mapping, which allows a quantitative assessment of diffuse cardiac fibrosis and estimations of the extracellular matrix volume (ECV) (Fig. 3) [85, 86]. There has been accumulating evidence that increased T1 times and ECV are related to clinical outcomes [87–89] and have potential for risk assessment in patients with AF.

## Conclusion

Heart failure is disease with large phenotypic variations in morphology, function and natural history. Such a heterogeneity in presentation is perhaps a reason why despite different clustering approaches, many interventions in clinical trials have not shown efficacy. Cardiac imaging provides diverse insights, but the ability to distinguish between overlapping phenotypes remains a challenging proposition. The evaluation of diastolic function by echocardiography and CMR with their traditional and novel techniques deserves specific analysis and a comparison with haemodynamic measurement before to be universally accepted. Diastolic function parameters derived by CMR may be applied in routine clinical care and matched with more feasible echo analysis in order to increase our awareness in the HFpEF mechanisms and reduces the current diagnostic gap. New parameters studying both radial and circumferential relaxation together with identification of extracellular collagen volume could facilitate the diagnosis and will play a central role in the identification of underlying pathophysiological mechanisms. Comparative imaging trials should be encouraged in order to discern which technique(s) alone, or in combination, could provide additional prognostic value.
